# Fall Prevention Knowledge, Attitude, and Practices of Community Stakeholders and Older Adults

**DOI:** 10.4061/2011/395357

**Published:** 2011-09-07

**Authors:** Sharon S. Laing, Ilene F. Silver, Sally York, Elizabeth A. Phelan

**Affiliations:** ^1^Health Promotion Research Center, Department of Health Services, School of Public Health, University of Washington, Seattle, WA 98105, USA; ^2^Senior Fall Prevention Program, Washington State Department of Health, Olympia, WA 98504, USA; ^3^Nursing and Healthcare Leadership Program, University of Washington, Tacoma, WA 98402, USA; ^4^Division of Gerontology and Geriatric Medicine, Department of Medicine and Department of Health Services, School of Public Health, University of Washington, Seattle, WA 98105, USA

## Abstract

We assessed knowledge, attitude, and provision of recommended fall prevention (FP) practices by employees of senior-serving organization and participation in FP practices by at-risk elders. The Washington State Department of Health administered structured telephone surveys to 50 employees and 101 elders in Washington State. Only 38% of employees felt “very knowledgeable” about FP, and a majority of their organizations did not regularly offer FP services. Almost half (48%) of seniors sustained a fall within the past 12 months; however, one-third perceived falling to be among their least important health concerns, and most had minimal working knowledge of proven FP practices. Seniors who perceived avoiding falls as important to their well-being were more likely to participate in practices about which they had the least knowledge (risk assessment, medication management). Increased awareness and availability of FP services might help engage older adults in FP practices and reduce the adverse effects of falls.

## 1. Introduction

Falls and fall-related injuries constitute an important public health concern. Each year, one in three community-dwelling older adults (65 years or older) sustains a fall [1, 2]. About 20–30% of falls result in serious injury, and injury care is costly [3–5]. As the aging population grows, the overall population burden will increase, and costs will rise substantially.

Falls are among the leading health indicators in *Healthy People 2020* [6], and several effective fall prevention (FP) practices have been documented for at-risk elders, including individualized fall risk assessment and multifactorial interventions [7, 8]. Little is known, however, about provision of FP services by community-based senior-serving organizations or about older adults' understanding of effective fall prevention practices. Understanding fall prevention knowledge, attitude, and practices (KAPs) of older adults and senior-serving organizations is crucial for translating and disseminating effective fall prevention programs. This study sought to obtain information about FP knowledge, attitude, and practices from employees of community-based organizations and from older adults at heightened risk for falling, in order to help establish a foundation for fall prevention initiatives in Washington State. The objectives of the current study were to ascertain (1) service providers' knowledge of, attitude about, and provision of practice-related services for senior fall prevention and (2) seniors' knowledge of, attitude about, and participation in recommended fall prevention practices. The long-term objective was to use the findings from the KAPs project to develop and enhance local programs, services, and educational materials; increase older adult access to programs; and build partnerships at the community level.

## 2. Materials and Methods

### 2.1. Setting

Trained interviewers administered structured telephone surveys to employees of senior-serving organizations in Washington State and older adults living in Pierce County, Washington. Both surveys were submitted to the Washington State Department of Social and Health Services IRB board and found to be exempt from the need for review.

### 2.2. Sampling

#### 2.2.1. Employees of Senior-Serving Organizations

**Figure 1:**
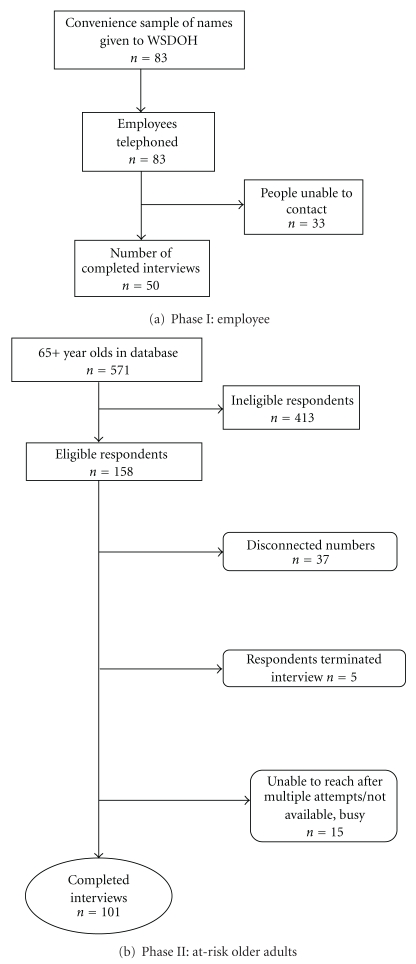
Recruitment of employees and older adults into the study.

The employees represented a broad range of urban and rural senior-serving and healthcare organizations in Washington State (see Table 1). The Washington State Department of Health (DOH) obtained the names of 83 employees who worked for organizations that serve older adults; Gilmore Research Group (a social marketing consulting firm in Seattle, Washington) successfully interviewed 50 among the 83 professionals (see Figure 1(a), Employee Recruitment Flowchart). The inclusion criteria for employee respondents were individuals from organizations offering services to and having regular contact with older adults. 

#### 2.2.2. Older Adults

Gilmore Research obtained a sample of telephone numbers from 571 households in Pierce County with residents aged 65 years or older, of which, 158 met the eligibility criteria. Eligible respondents were 65 years or older, lived in private/residential settings, and had experienced a fall or had concerns about falling. The screening for inclusion in the survey was, an affirmative response to any one of the following three criteria: (1) having fallen in the past 12 months; (2) restricting his/her usual activities because of fall-related fear; (3) having self-perceived balance/stability problems. Individuals unable to hear or understand verbal instructions due to language or cognitive difficulties were excluded. 

### 2.3. Measures

#### 2.3.1. Employee Questionnaire Development

The purpose of the structured employee telephone questionnaire was to assess employees' current knowledge of, attitude toward, and provision of best practices for preventing falls among older adults. The six fall prevention practices (described in Section 2.4) selected for the purposes of this study in 2004 were based on a review of the most current literature at that time which included the American Geriatrics Society's 2001 Clinical Guidelines for Prevention of Falls in Older Adults and two additional reports [9–11]. The DOH staff, Social Marketing Services Inc., and Gilmore Research Group developed the format and final questions for employee questionnaires, and Gilmore administered all interviews. The survey questions are available from the authors.

#### 2.3.2. Employee Questionnaire Administration

From July to August 2004, interviewers administered the structured telephone questionnaires to employees of senior-serving organizations. The questionnaire consisted of 24 open- and closed-ended questions. Interviewers began by informing respondents that the purpose of the interview was to obtain information to help reduce fall-related hospitalizations and deaths through community-based programming. The first few questions elicited responses about employees' perception of the urgency and preventability of falls, as well as their general knowledge about each of the six recommended fall prevention practices: individual fall risk assessment, strength/balance training, home assessment and safety improvement, medication review and management, training in assistive device, and fall prevention education (see Section 2.4 for brief descriptions). Interviewers then asked employee respondents about their perception of the importance of each FP practice, whether their organization provided FP services to elders, how often they offered FP services, and if they made referrals to other organizations offering FP services when such services were not available in their own organization. The interviewers ended the survey by asking employee respondents to list the main reasons that their organization did not offer one or more of the fall prevention best practices. Employee telephone interviews lasted approximately 15 minutes.

#### 2.3.3. Older Adult Questionnaire Development

The purpose of the older adult structured questionnaire was to assess elders' knowledge of, attitude toward, and participation in the six fall prevention best practices. Social Marketing Services and Gilmore Research Group drafted the instrument with refinements from DOH staff after pretesting the survey items on 12 community-dwelling older adults from the population of interest.

#### 2.3.4. Older Adult Questionnaire Administration

Interviewers administered the structured telephone questionnaires to older adults between December 2004 and January 2005. The instrument consisted of 52 closed-ended questions (not including eligibility screening items). The interviewers telephoned residents of selected households, requested permission to speak with one older adult available to be interviewed, and arranged a follow-up call for the unavailable respondent. Interviewers began the interview with the eligibility screening questions: whether the respondent had a recent fall, faced restriction in activity level due to fear of falling, and had balance or mobility problems. Interviewers then queried respondents about their perception of falls as a health and safety concern. Interviewers later assessed “unaided awareness” (no cues provided) by asking the respondent to state the activities that can prevent falls and evaluated “aided awareness” by asking respondents to state their awareness of the six fall prevention strategies presented to them. Subsequent questions listed each fall prevention strategy and asked the respondents their perception of the importance of the FP practice; their recent participation in the FP practice; the reasons for not participating in the FP practice (barriers); the factors likely to facilitate participation in the FP practice (motivators); resources that might increase older adults' knowledge about the FP practice. The final two questions queried older adult respondents on their understanding about how to learn more about fall prevention. The interviews lasted 15 to 20 minutes. 

### 2.4. Description of Key Variables—Fall Prevention Practices

The FP practices studied were (1) *individual fall risk assessment*: a health care professional, such as a doctor or nurse, conducting an assessment of fall risk and then providing recommendations on avoiding falls; (2) *strength and balance training*: training in special exercises to build strength and improve balance; (3) *home assessment and safety improvement*: assessing and modifying the home or having someone come into the home to demonstrate ways to protect against falling; (4) *medication review and management*: having a professional (i.e., physician/pharmacist) review medications that affect balance and help manage medications in order to prevent falls; (5) *training in assistive device (AD) use*: receiving special training from a physical therapist about how to use a cane or walker; (6) *fall prevention education*: receiving education that explains how to reduce fall likelihood.

### 2.5. Employee Response Items

The key variables assessed during interviews with employees were (1) *knowledge:* general knowledge of recommended fall prevention practices; (2) *perceived importance *(*attitude* **)**: assessment of the degree of importance of each FP practice; (3)* provision of services and referrals *(*practice*): the availability of fall prevention services, frequency of available services, and whether the organization primarily referred seniors to other places offering the FP service; (4) *barriers*: main reasons for not providing FP services.

### 2.6. Older Adult Response Items

Key variables assessed during interviews with older adult respondents were (1)(a) *unaided awareness *(*knowledge*): interviewers asked elders to verbally generate a list of potential activities to prevent a fall; (1)(b) *aided awareness *(*knowledge*): interviewers informed the respondents about each FP practice and asked about awareness of the practice; (2) *perceived importance *(*attitude*)*:* interviewers asked respondents about their perception of the importance of each FP practice; (3) *engagement/participation* (*practice*): interviewers discussed each best practice and asked about respondents' active participation in the practice; (4)* falls perceived as an important health issue*: interviewers asked respondents how concerned they were about falling in comparison to other personal health and safety issues; (5) *barriers*: interviewers asked respondents to think about a reason for not participating in each best practice; response options included “lack of awareness,” “no transportation,” “insufficient finances,” and “cultural barriers”.

### 2.7. Statistical Analyses

The dataset was deidentified prior to analyses and did not contain any linkages to respondents. The data were analyzed in 2005 using SPSS 10.0 (Chicago, IL). Percentages describe categorical data, and chi-square tests assess the significance of proportional differences. Unless otherwise noted, all reported statistically significant differences were calculated at the 95% confidence level. 

## 3. Results

### 3.1. Employee Characteristics

As seen in Table 1, fifty employees completed telephone interviews from a list of 83 names (62% response rate). The majority (54%) were professionals from senior-serving organizations, adult day health centers, senior centers, and emergency medical services. Employee characteristics data (e.g., professional background or length of time in present role) were unavailable for professionals representing senior-serving organizations. 

### 3.2. Older Adult Characteristics

Among the 571 households from the selected database meeting the age criterion, 158 met the eligibility criteria; 57 respondents were not able to participate for various reasons (see Figure 1(b) for older adult recruitment); a final sample of 101 older adult respondents completed the telephone interviews (64% response rate). Two-thirds of those interviewed were female (67%); most were over the age of 75 (60%); many were either married (50%) or widowed (38%) (Table 1). Almost one-half had sustained a fall in the past 12 months, and almost three-quarters (73%) limited their activity due to fear of falling. Two-thirds (65%) reported balance/mobility problems (Table 1).

### 3.3. Employee Fall Prevention Attitude, Knowledge, Practices, and Perceived Barriers

#### 3.3.1. Knowledge and Attitude

All employees identified falls to be an urgent (“very urgent” or “somewhat urgent”) health-related issue facing individuals aged 65 years of age or older, with 62% identifying falls to be a “very urgent” issue facing older adults. One-third (38%) felt “very knowledgeable” about recommended FP practices, and 58% perceived themselves to be “somewhat knowledgeable.” Two-thirds rated each of the prevention practices as “very important.”

#### 3.3.2. Practices

As seen in Table 2, strength and balance training and fall prevention education were the two practices that more than one-third (38%) of employees reported offering on a “regular” basis. For the other four fall prevention practices with low “regular” service provision (less than 20%), employees reported that their organizations offered fall prevention services “sometimes” or referred seniors to outside organizations.

Employees identified insufficient resources as the main barrier to regular provision of fall prevention services (80%); lack of funds was the primary resource limitation (66%). Other barriers included lack of trained personnel (28%), lower organizational priority (24%), and low awareness of the importance of fall prevention (22%).

### 3.4. Older Adult Fall Prevention Attitude, Knowledge, Practices, and Perceived Barriers

#### 3.4.1. Attitude

In response to the question about whether sustaining a fall represents a personal health and safety concern, one-third (34%) of elders reported falls to be one of their least important health concerns (Table 3(a)). Medication management, strength, and balance training, AD use training, and home safety were the fall prevention practices perceived as important by the largest proportion of seniors (65%, 59%, 49%, and 42%, resp.). Older adults perceived individual risk assessment and fall prevention education to be least important (29% and 22%, resp.), (Table 3(b)). 

#### 3.4.2. Knowledge and Practices

As shown in Table 3(b), unaided awareness of fall prevention best practices was generally low. Unaided, a larger proportion of seniors were more likely to name gait-related activities (moving slowly, wearing safe shoes, and using canes/walkers) and home safety improvement (69% and 21%, resp.). No seniors mentioned medication management unaided. However, seniors who perceived falls to be an important health concern were significantly more likely than seniors not viewing falls as important to engage in the practices about which they had the lowest unaided awareness: medication management, 66% versus 40%, *P* < 0.05, and risk assessment, 65% versus 30%, *P* < 0.05 (Table 3(c)).

When offered cues (aided awareness), seniors were most likely to show awareness of strength and balance training, home safety improvement, and medication management (88%, 61%, and 59%, resp.). Elders were least likely to show awareness of AD use training (27%). The prevention practices with the highest level of aided awareness were the fall prevention activities in which seniors were likely to participate (strength and balance training, 54%, and home assessment/safety improvement, 37%).

#### 3.4.3. Barriers

Older adults identified “not feeling at enough risk for falling” as the primary barrier to participating in most FP practices. They reported being *motivated* to actively participate in fall prevention practices when “something happened to increase their perception of risk,” or if they began “falling frequently”; these factors were the primary motivation for participation in three of the six fall prevention practices (medication review, home assessment, and individual risk assessment).

#### 3.4.4. Resources

Elders identified multiple sources for fall prevention information, with a significant majority preferring healthcare professionals (35%), followed by direct mailings (14%), and community centers (13%). Healthcare professionals were also viewed as the primary source of information about medications (78%) and AD use training (53%). Community/senior centers were identified as their primary information source for fall prevention education (20%) and strength and balance training (18%).

## 4. Discussion

Less than one-half (38%) of senior-serving professionals reported feeling “very knowledgeable” about recommended fall prevention practices; despite this, most perceived fall prevention to be important. Although a majority of senior-serving organizations did not offer “regular” FP services, more than two-thirds reported offering services “sometimes” or referred seniors to outside organizations; these actions suggest a willingness to address the health needs of seniors despite substantial barriers to implementing fall prevention programs. The evidence shows that senior-serving health providers can reduce fall likelihood by incorporating proven prevention strategies into clinical practice [12, 13].

Although almost one-half of older adults had a recent fall, one-third perceived falling to be one of their least important personal health and safety concerns. Further, they had minimal working knowledge (unaided awareness) of the proven measures to reduce fall risk. Of note, few older adults perceived individual fall risk assessment to be important, despite the strong evidence supporting this approach [7, 14]. Our data show that only 16% of senior respondents reported receiving individual risk assessments even though a substantial proportion had fallen (48%), which reinforces the importance of education about preventing falls through individualized treatment planning and longitudinal followup. Further, the updated American Geriatrics Society/British Geriatrics Society Clinical Practice Guideline calls for fall risk assessment not only for individuals reporting a fall but also for those demonstrating difficulty with gait or unsteadiness [14].

Others have noted that, although elders understand the importance of fall-related risk factors, they do not recognize their self-risk [15]; the lack of perceived risk for falling is an important barrier to senior participation in fall prevention programs [16], which suggests that, if seniors do not recognize their fall risk, they may be less likely to have a discussion with their physicians about how to reduce falls. Of note, these older adults preferred receiving FP information from a healthcare provider as their primary source of health-related information, a finding also observed by others [17].

Older adults did not have a high level of participation in any of the fall prevention practices with the exception of strength and balance training. However, it appeared that elders who perceived avoiding falls to be important to well-being were significantly more likely to participate in practices about which they had the lowest level of unaided awareness (individual risk assessment and medication management). This point is crucial, as it suggests that increasing understanding of the importance of fall prevention to health and safety may facilitate uptake of fall prevention practices. Fall prevention messaging targeting seniors must focus on the positive health and social benefits and be presented as life enhancing [16].

## 5. Implications

The current study contributes to the body of research that examines knowledge of, attitude toward, and participation in recommended fall prevention practices in community-dwelling older adults in the United States [15, 18, 19], as well as in Northern Europe, Australia, and New Zealand [20]. In one US study that examined how older adults prioritize competing health risks, fewer than 10% rated avoiding risk of fall injury to be of highest importance [18]. That work has shown that most elders do not perceive a personal fall risk even though they may understand that falls are preventable [13, 20]. Research has also shown that exposure to fall prevention initiatives does increase agreement that falls are preventable and raises fall prevention as a personal priority [20]. Knowledge of fall prevention practices and participation in those practices were not assessed in that study, and it is possible that heightened awareness about the adverse effects of falls and approaches to preventing a fall might affect prioritization [20]. The current study contributes to the literature on elders' attitude toward falls and prevention efforts by demonstrating the disparity that exists between the proven prevention strategies and elders' awareness and understanding of these practices.

Another primary contribution of the current study to fall prevention research is the insight offered about current service availability and service receipt for fall prevention best practices. Others have identified the fragmented nature of fall prevention care [21], but the current study may be the first to explore the availability of interventions in aging services in a particular geographic area and to query older adults about their perceptions and receipt of FP services prior to state-level dissemination of any fall prevention public health interventions. In our study, although a significant proportion of senior-serving organizations did not offer fall prevention services, research has shown that disseminating fall prevention evidence to clinicians and encouraging them to adopt fall prevention practices may reduce fall-related injuries and healthcare use for treatment of these injuries [22]. It is important therefore that proven fall prevention strategies are effectively translated for and disseminated to key stakeholders in order to raise awareness and encourage action. 

The current study also shows that for practices perceived by older adults to be important, ready availability of those fall prevention services may increase engagement likelihood, as evidenced by strength and balance training (59% of these community-dwelling older adults perceived strength and balance training as important, and 54% participated in the practice; also, 38% of senior-serving organizations offered strength and balance training regularly). Results of one other US study looking at receipt of physical activity programs showed contrasting outcomes [19]. In that study, nearly three-quarters of elders believed that physical activity was extremely important for fall prevention, but nearly one-quarter decreased their physical activity after a fall even with program availability. The authors explained the outcome by noting that a fear of falling can lead to the reluctance to adopt behaviors shown to prevent future falls [20].

## 6. Strengths and Limitations

A strength of the current work is that it may be the only study to provide a full evaluation of older adults' attitude toward, knowledge of, and participation in several recommended fall prevention practices. Previous studies have focused principally on older adults' attitude and queried older adults about a smaller number of recommended FP practices [23]. Information about elders' knowledge of, attitude toward, and participation in the six different FP practices presented in the current study offers a broader assessment and may guide program planners to develop targeted messages about each fall prevention practice, particularly for those about which elders have less knowledge.

A second strength is that the study provides information about FP knowledge of employees of senior-serving organizations and quantification of offered FP services, prior to the development of statewide fall prevention initiatives. This information may help to direct efforts to broadly disseminate fall prevention strategies to community-based organizations and permit assessment of progress over time.

The study also has a few limitations. Results may not generalize to all elders in the US due to the restrictive nature of the sampling. The older adult sample included individuals reachable by telephone and excluded residents of long-term care facilities. It is important to note, however, that there are no reliable figures on the incidence of fall-related concerns among older adults in Washington State, and, therefore, the data can potentially provide good baseline information about knowledge, attitude, and practices of a sample of community-dwelling older adults in the region.

Limitations with respect to the employee survey include the fact that survey items were not pretested and that selection bias may have been present. A few of the representatives were aware of the DOH Injury and Violence Prevention Program's interest in addressing older adult fall prevention; therefore, some respondents may have been motivated to give favorable representations of their organizations and, also, for social desirability purposes, to endorse fall prevention as a critical health issue. It is important to note that overall service provision was low and may, in fact, have been lower had the study randomly sampled senior-serving organizations.

A final limitation is the fact that the project was conducted six years earlier, and therefore changes may have occurred in the knowledge, attitude, and practices of older adults and senior-serving organizations during the intervening period. It is important to reassert that a value of the study is that information about baseline fall prevention KAPs in Washington State has the potential to augment fall prevention efforts at the state and local public health departments. 

## 7. Conclusions

Clear fall prevention messaging targeting older adults and providers appears warranted. Messages targeting senior-serving organizations should focus on increasing awareness of specific fall prevention practices shown to be effective in reducing falls. Messages targeting elders should address the importance of fall prevention for older adult health, educate about specific FP practices and emphasize the importance and effectiveness of fall prevention strategies for preserving function, independence, and well-being.

##  Authors' Contribution

I. F. Silver and S. York, contributed in concept and design, acquisition of data, data analysis and interpretation, and paper preparation. S. S. Laing and E. A. Phelan contributed in data analysis and interpretation and manuscript preparation.

##  Disclaimer

Centers for Disease Control and Prevention played no role in the design, methods, subject recruitment, data collection, analysis, or preparation of the paper. The contents of this paper are solely the responsibility of the authors and do not necessarily represent the official views of the Washington State Department of Health or the Centers for Disease Control and Prevention.

## Figures and Tables

**Table 1:** Demographic characteristics of older Adults and organizational characteristics of senior-serving employees in Washington State, 2004.

Senior clients' demographic characteristics, *n* = 101	**%**
*Gender*	
Male	33
Female	67
*Age*	
65–74	41
75–84	48
85+	12
*Income level*	
<$25,000	40
$25,000–49,999	44
>$50,000	16
*Marital status*	
Married	50
Single	9
Widowed	38
Divorced	3
*Living status*	
Live in home/apartment	98
Live in assisted living facility	2
*Reports of balance, falls and other mobility problems*	
At least 1 fall in last 12 months	48
Limiting activity due to fear of falls^†^	73
Balance and/or mobility problems	65

Organizational characteristics of key informants working in senior-serving agencies, *n* = 50	**%**

*Types of organization: community/aging services*	
Senior service (professional/experts)	18
Adult day health	12
Senior center	12
Area Agency on Aging	8
Residential living facility	8
AARP	4
Meals on Wheels	4
*Types of organizations: health care system*	
Emergency Medical Services	12
Public health department	8
Hospital-based older adult program	6
Home health agency	4
Community clinic	1
ED/Trauma service	1
Fall prevention program	1
Geriatrician	1

^†^27% of older adults use canes/walkers (see Table 3(b)).

**Table 2:** Attitudes and provision of fall prevention services among community-based, senior-serving organizations, *n* = 50.

Fall prevention practice	Practice perceived as very important	Provision of service on a regular basis*	Provision of service sometimes*	Referral to outside organization(s) to provide service*
		**%**		

Individual assessment of risk	74	16	36	26
Strength and balance training	94	38	28	24
Home assessment and safety improvement	76	14	34	40
Review and management of medications	84	10	22	44
Training and use of assistive devices	68	8	26	42
Fall prevention education	74	38	30	20

*Provision of services is captured in the last three columns of this table. By summing the values in the last three columns, the total service offered for each FP practices is obtained. Therefore, for *Individual Risk Assessment*, 78% of senior-serving organizations addressed this practice by offering services regularly, sometimes or referring older adults to outside organizations (16% + 36% + 26% = 78%).

**Table:** (a) Older adults concern about falling compared to other personal health and safety issues, *n* = 101

Fall concerns	**%**
One of the most important health and safety issues that concerns you	31
Only one of several you are concerned with	36
One of the least important personal health and safety issues you are concerned with	34

**Table:** (b) Older adult knowledge of, attitude toward, and participation in, fall prevention practices, *n* = 101

Fall prevention practice	Endorsed when unaided	Endorsed when aided	Practice perceived as very important	Participation in practice

		**%**		

Individual assessment of fall risk	1	52	29	16
Strength and balance training	8	88	59	54
Home assessment and safety	21	61	42	37
improvement				
Review and management of medications	0	59	65	29
Training and use of assistive devices*	69^†^	27	49	48
Fall prevention education	2	34	22	4^‡^

*Only 27% of older adults used canes or walkers.

^†^Although specific training in using walkers and canes was not mentioned by any of the respondents, they were focused on being cautious when walking, moving slowly, wearing good/safe shoes, using canes/walkers, and holding on to things.

^‡^Indicates past year participation in fall prevention education.

**Table:** (c) Older adults' participation in practices based on the perceived importance of falls as a health concern, *n* = 101

	Falls perceived to be an important health			
	and safety concern			
Fall prevention practice	Yes		No	
	*n*	(%)	*n*	**(%)**

Individual assessment of risk				
Participated in practice	20	(65)	21	(30)*
Not participated	11	(35)	49	(70)
Strength and balance training				
Participated in practice	19	(61)	53	(76)
Not participated	12	(39)	17	(24)
Home assessment and safety improvement				
Participated in practice	26	(84)	46	(66)*
Not participated	5	(16)	24	(34)
Review and management of medications				
Participated in practice	19	(66)	27	(40)*
Not participated	10	(34)	40	(60)
Training and use of assistive devices^†^				
Participated in practice	6	(55)	8	(44)
Not participated	5	(45)	10	(56)
Fall prevention education				
Participated in practice	5	(16)	10	(14)
Not participated	26	(84)	(60)	(86)

*Significant *P* < 0.05 (*P* = 0.05 is not statistically significant).

^†^Only 27 persons reported using a cane/walker.
